# Is home-based management feasible for COVID-19 patients requiring oxygen therapy? A participatory action study and cost analysis

**DOI:** 10.1136/bmjopen-2025-113288

**Published:** 2026-09-03

**Authors:** Josi A Boeijen, Alma C van de Pol, Karin Smit, Rick T van Uum, Abeer Ahmad, Miriam P van der Meulen, Robert van den Broek, Wilma G Bijsterbosch, Roderick Venekamp, Frans H Rutten, Dorien LM Zwart

**Affiliations:** 1 Julius Center for Health Sciences and Primary Care, Utrecht, The Netherlands; 2 Department of General Practice and Nursing Science, Julius Center for Health Sciences and Primary Care, Utrecht, The Netherlands; 3 Department of Epidemiology and Health Economics, Julius Center for Health Sciences and Primary Care, Utrecht, The Netherlands; 4 Department of Internal Medicine and Acute Medicine, University Medical Centre Utrecht, Utrecht, The Netherlands

**Keywords:** General Practice, COVID-19, Oxygen Saturation, Respiratory infections, Hospital to Home Transition

## Abstract

**Objectives:**

To explore feasibility and estimate cost of a home-based intervention for COVID-19 patients requiring oxygen therapy.

**Design and setting:**

Participatory action study embedded in a regional collaborative healthcare network.

**Participants:**

13 patients were included (77% female; mean age 68 years, SD 18.2).

**Intervention:**

A previously designed intervention was piloted and iteratively evaluated in a multidisciplinary expert panel using a participatory action research approach.

**Primary and secondary outcome measures:**

We scrutinised clinical course data and monitored adverse events. Feasibility was assessed using 12 key elements related to (1) communication, (2) logistics, (3) treatment and remote monitoring and (4) adverse events. In an explorative cost analysis, intervention costs were compared with hospital admission costs as the reference.

**Results:**

Home-based management lasted for 7 days (SD 3.4) on average, with 4 days (SD 2.6) oxygen therapy. No intervention-related adverse events occurred. Main challenges were related to interprofessional communication, nursing care organisation and timely delivery of oxygen concentrators. Excluding costs for setting up the intervention, costs were estimated at €1124 per patient and this was mainly driven by costs on the first day. Compared with hospital admission, the cost-saving potential is estimated at €1087 to €2850 per patient.

**Conclusions:**

An acute home-based care intervention embedded in a regional collaborative care network for patients with acute respiratory tract infections requiring oxygen therapy seems feasible and is potentially cost-saving.

**Trial registration number:**

The Dutch Trial Register CCMO: NL77421.041.21; OMON Register: NL-OMON22655.

**STRENGTHS AND LIMITATIONS OF THIS STUDY**

- The generic intervention design makes the building blocks applicable to other settings and diagnoses, even beyond the COVID-19 pandemic.
- Intervention evaluation was done in a multidisciplinary expert panel, including patient and professional views.
- 13 detailed case studies yielded important information on feasibility.
- Cost analysis was explorative due to retrospective data collection, but provides valuable insight into total potential cost-savings and into elements that contribute to costs.
- Since only 10 patients fully received the intervention, safety data should be interpreted cautiously.

## Introduction

 Home-based instead of hospital-based care is associated with lower rates of hospital-associated complications, better patient and family member care experience and lower costs.^1–6^ Spurred by the COVID-19 pandemic, along with the anticipated shortage in healthcare workforce capacity, novel approaches to deliver safe and patient-centred care are prioritised.^7–11^ Home-based instead of hospital-based management of patients who require oxygen therapy due to community-acquired acute respiratory tract infections (CA-ARTIs), such as COVID-19, is such a novel approach.

Initially, many new initiatives focused on surveillance of patients with mild disease symptoms at home.^12–14^ Home-based monitoring of pulse oximetry in patients with mild symptoms has been proven feasible and reassuring for patients with CA-ARTIs.^15^ Adherence to a self-measurement regimen was high and patients reported a high feeling of safety.^15 16^

For patients with severe disease symptoms, many studies describe successful early hospital discharge initiatives, where patients recovered at home with supplemental oxygen therapy after initial hospitalisation.^3 17–22^ Reports on acute home-based management programmes for patients requiring oxygen therapy, that is, without initial hospital admission, are scarce.^23–26^

Studies thus far mainly focused on home-based management of patients who suffered from mild COVID-19 symptoms with only a minority receiving oxygen therapy, leaving the safety, feasibility and notably the cost-saving potential of a complete acute home-based intervention for patients with CA-ARTI uncertain.

We therefore conducted a pilot study to explore safety, assess feasibility and estimate cost of a previously designed home-based management intervention for COVID-19 and other CA-ARTIs patients requiring oxygen therapy. The intervention consists of five components (‘building blocks’): (1) acute medical care; (2) organisation and logistics; (3) remote monitoring; (4) acute nursing care; (5) equipment and technology. It was designed in a multidisciplinary expert panel using a participatory action research approach.^27^

## Method

### Design

The design of the intervention has been published previously.^27^ Following a participatory action research approach, the intervention was piloted and iteratively evaluated in a multidisciplinary expert panel that assessed 12 predefined key elements related to safety and feasibility.

#### Participatory action research approach

Participatory action research (PAR) is a research process which requires active involvement of different stakeholders. PAR aims to (1) trigger a change process for solving a practical problem, (2) be a learning process among those directly involved and (3) help further develop scientific knowledge. It simultaneously facilitates change in daily practice and contributes to the scientific debate. The PAR approach works through an iterative process of planning, action and reflection together with those who will be directly involved in executing the intervention.^28^

#### The multidisciplinary expert panel

The multidisciplinary expert panel was involved in the design, development and evaluation of the intervention throughout. The panel was recruited by the research team from relevant stakeholders in the region.^27^ The panel consisted of general practitioners (GPs) from four regional primary care groups; pulmonologists and acute care internists from four hospitals in the region; home care nursing organisations and an office for nursing care mediation; a monitoring centre; regional care coordination centre; and a patient representative from the Utrecht Elderly Care Network.

### Setting

Participants were included from November 2022 until December 2023 in Utrecht, the Netherlands. Participants were representative for the Dutch population and included participants from urban and rural areas. The intervention was embedded within a regional collaborative care network.

### Participants

Inclusion and exclusion criteria were described previously^27^ and in detailed in online supplemental table 1. In short, adults with COVID-19 or CA-ARTI requiring oxygen therapy (peripheral oxygen saturation (SpO_2_) <94%) and/or respiratory distress (respiratory rate >24 breaths per minute) were eligible. This included two types of patients (type I and II), with minor differences in intervention protocol (online supplemental figure 1). Patients who required more care than possible at home were excluded, for example, patients with haemodynamic instability or oxygen need >5 L/min.

#### Informed consent procedure

Written informed consent was obtained from all participants at home or at the emergency department (ED).

### Intervention

**Figure 1:**
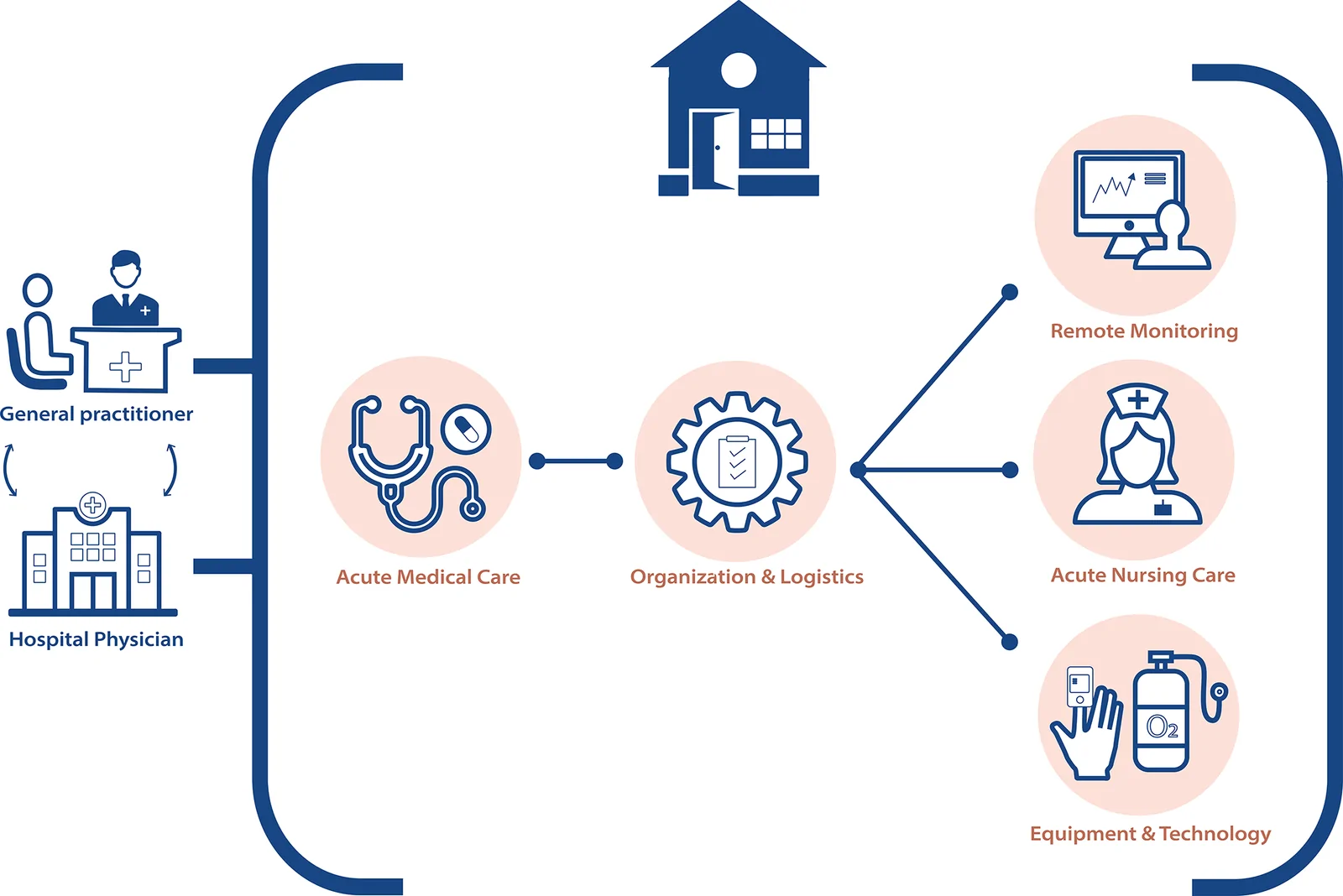
Five ‘building blocks’ of the intervention. (1) Acute medical care: the GP or hospital physician initiates the intervention and provides acute care and the treatment plan. Oxygen therapy titration protocols were standardised, but individual care plans could vary. For patients with COVID-19, a regimen of dexamethasone and thrombosis prophylaxis was recommended. (2) Organisation and logistics: a regionally available care coordination centre organised acute nursing care, expedient delivery of equipment, and activation of the monitoring team. (3) Remote monitoring: Three times daily the patient registered vital parameters (peripheral oxygen saturation, heart rate, temperature) and a shortness of breath score (Visual Analogue Scale 1–10). Once daily, patients registered additional scores for cough (VAS 1–10) and general well-being. The monitoring team had contact with the patient through an app (Luscii Healthtech BV) and/or via phone. (4) Acute nursing care: a specialised home care nurse provided in person clinical evaluation of the patient on day 0, 1 and 2. (5) Equipment and technology: An oxygen concentrator and standard nasal cannula (supplying a maximum of 4–6 L/min) were delivered and installed at home expediently (within 4 hours). Patients were provided with a pulse oximeter validated for medical use and a standard ear thermometer. GP, general practitioner; VAS, Visual Analogue Scale.

The intervention consists of five components (‘building blocks’): (1) acute medical care; (2) organisation and logistics; (3) remote monitoring; (4) acute nursing care; (5) equipment and technology (figure 1). The intervention was previously described in more detail.^27^

### Data collection and analysis

Data on safety, feasibility and cost were collected during a 30-day follow-up period.

#### Safety

##### Data collection

Patient characteristics, symptomatology at inclusion and healthcare use data were extracted from the patients’ GP electronic medical records (EMR). Clinical course data (vital parameters and symptom scores; online supplemental table 2) were recorded by the monitoring team during remote monitoring contacts. Occurrence of serious adverse events,^29^ such as hospital admission or death, were reported directly to the research team by the involved healthcare professionals. The patients’ GP EMR was reviewed for missed (serious) adverse events. WHO Disability Assessment Score 2.0 (WHODAS 2.0)^30^ data were collected by phone or visit on day 0 and 30. Patients scored their ‘feeling of safety’ on day 14 on a Visual Analogue Scale (0–100; 0=extremely unsafe, 100=fully safe).

##### Data analysis

The expert panel explored safety issues during short cycle (biweekly) and large cycle (every 6–8 weeks) evaluation rounds by scrutinising safety data. During short cycle evaluations the panel evaluated whether safety issues had occurred and if so, whether these were attributable to intervention elements. Subsequently, the panel established whether it was safe to proceed, or if any intervention adjustments were required. During large cycle evaluations, data from all included patients were re-evaluated.

### Feasibility

#### Data collection

We collected communication data through structured evaluation forms for patient/informal caregiver and all involved healthcare professionals. Logistics data were collected through evaluation forms for the care coordination centre, nursing care organisation, oxygen supplier and complemented with observations of the coordinating researcher.

#### Data analysis

**Figure 2:**
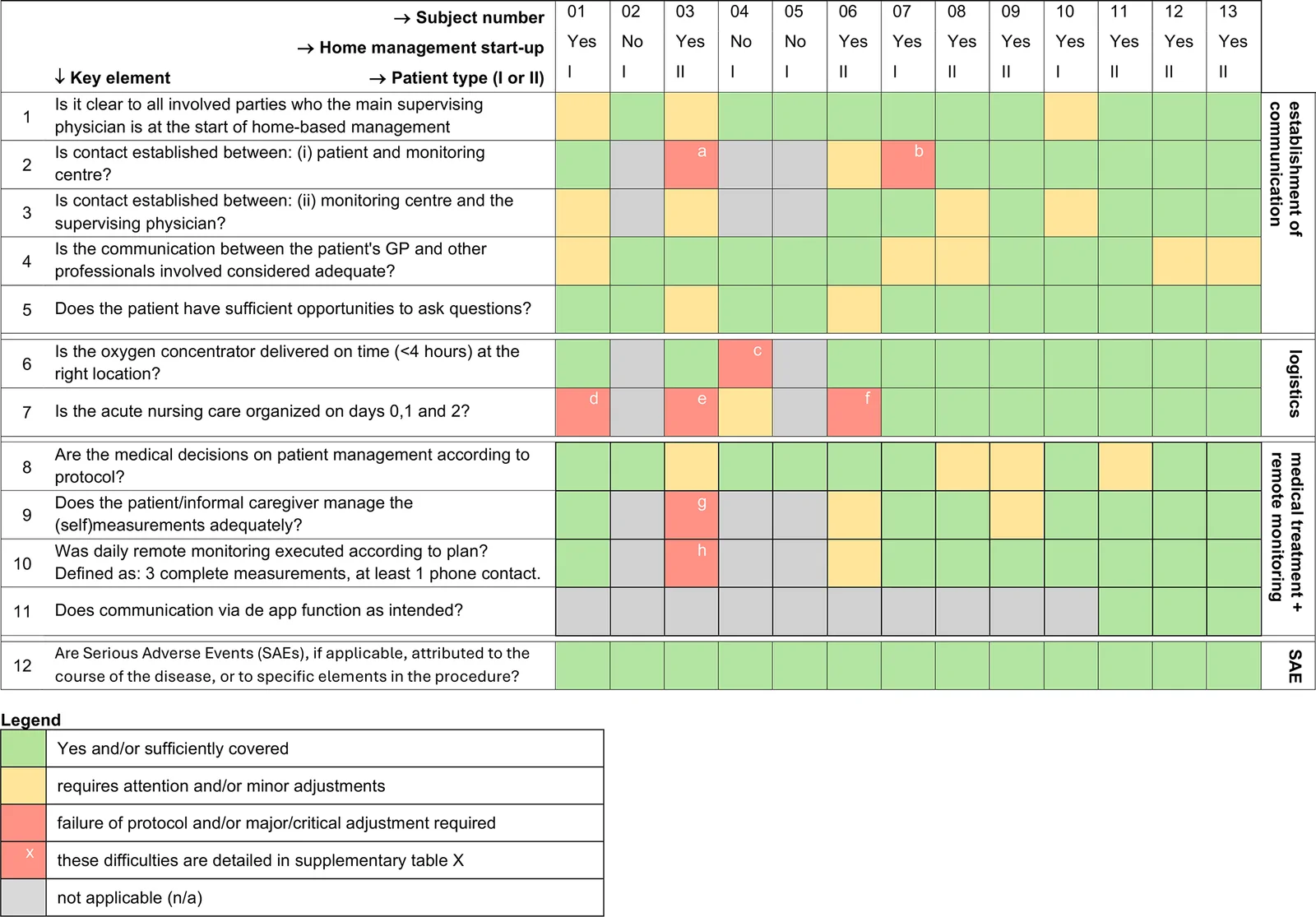
Overview of the intervention’s ‘key elements’ and the scoring results. GP, general practitioner.

Feasibility was evaluated by scoring the predefined ‘key elements’.^27^ The key elements were related to four topics: (1) establishment of communication (key element 1–5), (2) logistics (key elements 6 and 7), (3) medical treatment and remote monitoring (key elements 8–11) and (4) serious adverse events (key element 12; figure 2). Scoring options were green (‘yes/sufficiently covered’); yellow (‘requires attention and/or minor adjustments’); red (‘failure of protocol and/or major/critical adjustment required’); or grey (‘not applicable’). In the short cycle evaluations the research team completed a preliminary scoring based on the available data at that time. During the large evaluations the panel adjusted scores if needed.

### Explorative cost analysis

We performed an explorative cost analysis evaluating the set-up costs and per patient intervention costs using a mixed-method approach. As data collection was retrospective, we did not perform a full health economic evaluation, but rather a cost calculation of the intervention itself. To illustrate potential cost-savings, we did a comparison to reference costs of a hospital admission. The estimation of the set-up costs was based on expert opinion (online supplemental table 3).

Per patient intervention costs were estimated in three steps: (1) we identified cost-components with two semi-structured interviews with the main project leaders; (2) we performed expert consultations with one stakeholder of each cost-component to validate the cost-components, identify methods to calculate costs and perform expert opinion on the costs; (3) we performed mixed-methods calculations for each cost-component. Identified cost-components and the results of steps 1 and 2 are listed in online supplemental table 3. The results of step (3) are presented in the results. Total intervention costs were compared with the costs of an admission day in the hospital multiplied by the duration of admission. The duration of admission was estimated in two scenarios, one assuming duration of admission to be equal to the length of days on oxygen and one assuming duration of admission to be equal to duration of the telemonitoring. In this analysis, we assumed non-inferiority in all costs after the admission. We, for instance, did not assume a difference in readmission rates following hospital discharge or acute home-based management.

### Patient and public involvement

Patient representatives were involved in all stages of our pilot. A patient representative from the Utrecht Elderly Care Network helped with designing, formalising and continuously evaluating the intervention. She was also a participant in the multidisciplinary expert panel. We had a simulation patient as a test case to trial the intervention logistics prior to the inception of the pilot. Furthermore, the experience of patients participating in the pilot was used in the iterative PAR approach in our pilot study to further develop the intervention. For this, we used patients’ experiences as collected from both questionnaires and qualitative interviews.

## Results

### Participants

**Figure 3:**
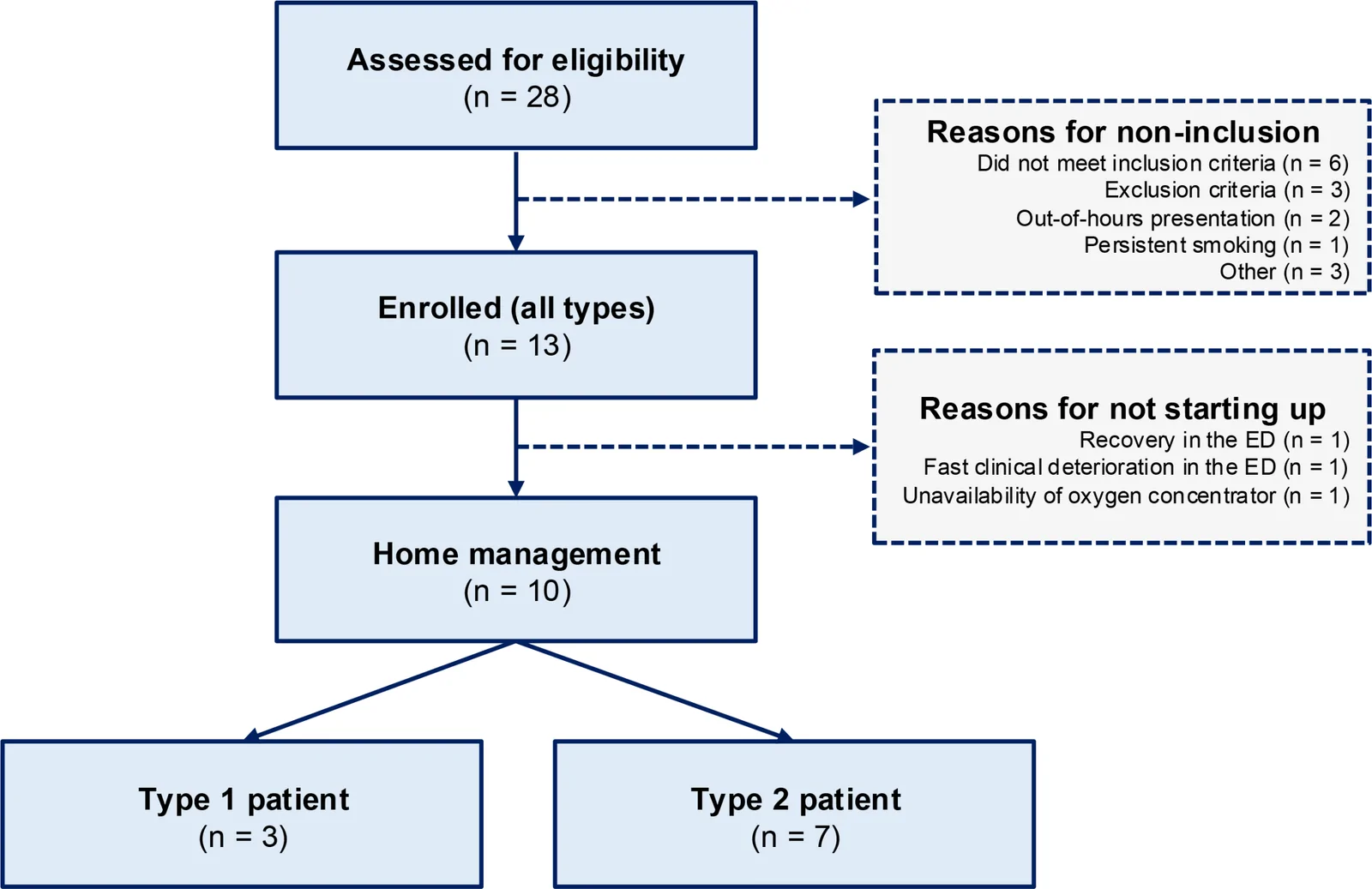
Participant inclusion flowchart. ED, emergency department.

Of the 28 patients assessed for eligibility, 13 were included (figure 3). Enrolled patients had a mean age of 68 years (SD 18) and 77% were female (table 1). Two patients lived in a care facility (15%) and the remaining in a private residence 23% of patients had no comorbidities; 38% had >2 comorbidities. Nine patients were hypoxaemic at inclusion (69%), and four had an increased respiratory rate (31%).

**Table 1:** Participant characteristics (n=13)

Mean age in years (SD)	68 (18)	
Medical history and comorbidities*	n	%
Gender		
Female	10	77
Male	3	23
History of TIA/stroke	2	15
History of ACS	2	15
Diabetes mellitus type II	3	23
BMI >30 kg/m^2^	2	15
COPD gold I-II	3	23
COPD gold III-IV	1	8
Chronic kidney disease	1	8
Hypertension and/or hypercholesterolaemia	6	46
Heart failure	1	8
No comorbidities	3	23
≥2 comorbidities	5	38
Patients using chronic medication	6	46
Smoking		
Current	1	8
Former	3	23

Clinical status on day 0 reported by GP and/or ED physician		
Number of days with symptoms		
1–4 days	4	31
5–8 days	3	23
9–12 days	6	46
SpO_2_		
≥94%	4	31
90–93%	5	38
86–89%	4	31
Respiratory rate >24 breaths per minute†	4	31
Tachycardia >100 beats per minute†	2	15
Temperature >38°C†	1	8
Preliminary diagnosis		
COVID-19	10	77
Exacerbation COPD triggered by COVID-19	2	15
Exacerbation COPD and influenza-like symptoms	1	8

* Data incomplete. No access to GP electronic patient file in 2/13 patients; history and comorbidity data could not be verified.

† Data incomplete. Respiratory rate not reported in 3/13 patients, heart rate not reported in 4/13 patients, temperature not reported in 6/13 case. Calculation of percentage assumes values that were not reported were not above the cut-off point.

ACS, acute coronary syndrome; BMI, body mass index; COPD, chronic obstructive pulmonary disease; ED, emergency department; GP, general practitioner; SpO_2_, peripheral oxygen saturation; TIA, transient ischaemic attack.

10 participants received the full intervention (3 type I patients, and 7 type II patients). Three participants did not receive the intervention due to: (1) sufficient recovery in the ED (2) fast clinical deterioration necessitating immediate hospital admission, (3) unavailability of oxygen concentrators.

#### Clinical course data

Home-based management lasted for 7 days (SD 3.4) on average, with a mean of 4 days (SD 2.6) oxygen therapy. Medical treatment details are shown in online supplemental table 4. Clinical deterioration (eg, SpO_2_ drops below 94%) occurred in two type II patients. One of these patients remained at home with adjustment of oxygen supplementation and additional follow-up contacts with the monitoring team and out-of-hours GP service. The other patient was admitted to the hospital after 6 hours of home-based management and died 8 days later (diagnosis: chronic obstructive pulmonary disease (COPD) exacerbation, COVID-19 and bacterial superinfection).

#### Safety

The multidisciplinary expert panel did not encounter indications of unsafety during the short or large cycle evaluations. One serious adverse event occurred in a type II patient (hospital admission and subsequent death). The patient’s clinical deterioration and subsequent hospital admission occurred within 6 hours of home-based management initiation. Care escalation was evaluated by the panel as timely and adequate. The adverse event was therefore attributed to the course of the disease, and not to the intervention.

On day 14, patients reported a mean ‘feeling of safety’ score of 83 (SD 9). After 30 days, patients reported a 54% reduction in disability as measured with WHODAS 2.0 compared with day 0 (online supplemental table 5).

### Feasibility

An overview of key element scoring is provided in figure 2.

#### Communication (key element 1–5)

Communication between professionals in the collaborative care network faced challenges, particularly at the start and end of the intervention. Patient information was mostly exchanged via phone or secure email, which was inefficient and due to separate electronic files kept across care providers GPs, hospital specialists, home care nurses, care coordination centre and monitoring staff.

Initial (day 0) establishment of communication between patient and monitoring centre was inadequate in two instances (online supplemental table 6a, b). In the first instance, the informal caregiver unexpectedly refused phone access to the patient (extenuating circumstance). This instance underpinned that both patient and informal caregiver have to be well-informed and cooperative during home-based management. In the second instance, the monitoring centre had been unaware of the inclusion because the care coordination had forgotten to inform them (human error). Both instances identified challenges that were eliminated by additional training of personnel.

A learning curve was evident (figure 2), with improved feedback loops and familiarity among professionals, though some challenges persisted.

#### Logistics (key elements 6 and 7)

Logistics were delegated to a centrally organised care coordination centre. In the first six patients logistical challenges occurred (figure 2). Expedient (<4 hours) oxygen concentrator delivery was obstructed once due to concentrator shortage, and once because evening delivery was unavailable. Acute nursing care was not possible due to personnel shortage in one case and financing issues in another. Bottlenecks in the logistics were largely resolved by introducing of a brief (<24 hours) hospital admission option, mainly to facilitate organisation of logistics in the evening hours.

#### Treatment and remote monitoring (key elements 8–11)

In key element 8 we evaluated whether the predefined treatment protocol was adequately used by physicians. One GP indicated that, in addition to the online protocol, training in oxygen therapy would have been useful. Several (minor) deviations from protocol occurred. In one case, for example, the monitoring team had registered a decline in SpO_2_, but subsequent ED evaluation failed because the patient refused the ambulance ride.

In key elements 9–11, various aspects of remote monitoring were assessed. Patient self-measurements and reporting of symptoms was done according to protocol in most cases. In a single case, the patient and informal caregiver indicated that the monitoring routine was too elaborate for them to manage.

Monitoring contacts via phone or app were both evaluated as effective by patients and the monitoring team. Four patients had a strong preference for phone contact and would not have participated if remote monitoring was only offered via app. The monitoring staff indicated that fewer contact moments were needed when patients were in recovery.

GP consultation workload is reported in online supplemental table 7. A median of 4 (IQR 3–6) GP consultations were registered per patient. Most consultations were conducted via phone (71%).

### Explorative cost analysis

Total costs were divided in set-up costs and per patient intervention costs (online supplemental table 3). Set-up costs include (1) personnel costs for development of the app (€14 000 to €22 000), (2) costs for licensing of the app (€8000 to €30 000, depending on already existing licensing) and (3) costs for the infrastructure (€12 500 to €20 000).

Per patient costs of the intervention are presented in table 2 and detailed in online supplemental table 3. First day costs have a large contribution to total costs due to ambulance transport, oxygen equipment delivery and several intakes.

**Table 2:** Estimated costs per cost-component of the intervention on the first day and all subsequent days, compared with two scenarios of hospital admission costs

First day costs	
Ambulance ride	€273
Care coordination	€38
Intake at emergency department	€90
Oxygen delivery	€250
Home care intake	€186
Monitoring centre intake	€12
Costs in the subsequent days	
Home care	€105
GP visits	€148
Monitoring centre	€23
Total costs	
Total per patient costs of the intervention	€1124
Cost comparison	
Total patient hospital admission of 4.1 days (based on length of oxygen therapy)	€2202
Total patient hospital admission of 7.4 days (based on length of remote monitoring)	€3974

GP, general practitioner.

Per patient intervention costs could amount to €1124. As a comparison, based on standard unit costs, a hospital admission costs €2202 if patients are admitted 4.1 days (in line with the average duration of oxygen therapy) or €3974 if patients are admitted 7.4 days (in line with the average length of remote monitoring), resulting in potential cost-savings of €1087 to €2850 per patient. With a cost-saving estimate of €1087 to €2850, and high set-up costs of €34 500 to €72 000, 13–67 patients should undergo acute home management for CA-ARTI before the break-even point is reached.

## Discussion

The cases of patients with CA-ARTI requiring oxygen therapy that we extensively evaluated in this pilot indicate that an acute home-based intervention embedded in a collaborative care network is feasible and potentially cost-saving.

### Strengths and limitations

A key strength of this study is the iterative evaluation with a multidisciplinary expert panel, including patients and professionals. We also aligned the intervention’s components with available digital and human resources to support practical implementation. Lastly, the generic design makes the building blocks applicable to other settings and diagnoses, beyond the COVID-19 pandemic.

Some limitations deserve further attention. First, since only 10 patients fully received the intervention, the clinical course and safety data should be interpreted very cautiously. Nonetheless, we believe the 13 detailed case studies yielded important information on feasibility. Second, our predefined key elements may not be fully transferable to other contexts as the design of our collaborative network intervention remains at least partly context-related. Third, the cost analysis was an explorative calculation of the intervention itself. It was not possible to perform a full health economic evaluation due to retrospective data collection. Still, the results provide insight into total potential cost-savings, and into elements that contribute to costs. Moreover, the explorative cost calculation can guide design of a detailed prospective cost analysis of similar interventions.

### Comparison with existing literature

#### Home-based management within a collaborative care network

Setting up an inter-organisational, collaborative care intervention is laborious. Similar home-based interventions from previous studies were carried out by a single organisation,^25 26 31–33^ most often a hospital. We deliberately engaged existing regional healthcare providers in a collaborative network—a community of practice^34^—for two main reasons. First, co-creating with a committed coalition helps prevent healthcare fragmentation and values each provider’s expertise, fostering shared ownership. Second, this approach promotes efficient use of human and digital resources, enhancing sustainability amid rising costs and workforce shortages.^35–38^ We believe this collaborative approach offers a more scalable and sustainable path to acute care transformation than single-provider models.

A study examining inter-organisational collaborative care argued that effective collaboration is developed through three processes: (1) redefining ‘areas of responsibilities’ per organisation; (2) reconstructing the ‘ways of working’; and (3) organising a networking model with the patient at the centre.^39^ The third process proved most complex, as effective communication between professionals of various organisations was challenging. In line with well-known hazards of inter-organisational handover,^40–43^ we observed the risk of task and responsibility mix-up between professionals despite thoroughly defining responsibilities. These challenges may easily introduce misunderstanding, leading to suboptimal care. We suggest two possible solutions. First, allowing healthcare professionals to work in universal, inter-organisational, privacy proof communication portals would be helpful, but these portals are currently lacking. Second, in acute hospital settings, crew resource management training has been shown to improve teamwork and effective handover communication.^44 45^ Both may help to improve effective collaboration within a care network.

#### Home-based management during the COVID-19 pandemic

Several observational studies suggest that home-based instead of hospital-based management may be feasible for COVID-19 patients, and reported preliminary data on safety.^24–26 46^ Three studies based in the USA and Spain focused on patients with mild COVID-19 who would, in USA and Spanish usual care, be admitted to the hospital. Of these cases, however, only a minority required oxygen therapy. Of all enrolled patients (n=684) in these studies, 6–21% of patients were admitted to the hospital ward during home-based management, and 0−8% of patients died. A fourth, UK-based study described an intervention for frail COVID-19 patients who were unlikely to benefit from hospital care, similar to type II patients in our pilot study.^46^ Of the severe cases requiring oxygen treatment (n=33), 9% were admitted to the hospital, and 18% died. Although the varying case-mix of patients hampers direct comparison, these studies indicate that home-based interventions delivering hospital level care have been feasible in other contexts as well.

### Implications for research and practice

Importantly, formal safety and clinical-effectiveness of home-based management for patients with CA-ARTI needing oxygen remains unproven. Robust evidence from a well-powered pragmatic randomised controlled trial is urgently needed to guide safe, effective decision-making for both clinicians and policymakers. This is especially relevant in healthcare systems with strong primary care and strict gatekeeping, where patients with CA-ARTI may present for hospital admission with more severe illness.

The exploratory cost analysis identified set-up and first-day costs as the main cost contributors. High set-up costs suggest that sufficient patient volume is needed to achieve return on investment. Per-patient costs were largely driven by high first-day expenses, making longer home-based care more cost-efficient per day. Thus, greater savings are expected in case of large patient volume and for patients requiring prolonged home-based management.

Further qualitative research is needed to identify the barriers and facilitators that influence healthcare professionals’ willingness to participate in home-based management programmes. Concerns about increased workload are frequently cited, yet our experience suggests that, with well-organised logistics and central monitoring of oxygen saturation and clinical symptoms, the additional burden on both hospital physicians and GPs may be kept to a minimum.

Known challenges in CA-ARTI management, such as strain on healthcare systems due to seasonal peaks and the lack of tailored care pathways minimising hospital admissions for elderly patients, existed before the pandemic and will persist afterwards. Beyond the COVID-19 pandemic, integrating home-based management interventions into standard care pathways may provide the necessary healthcare system flexibility to face these known and future challenges in CA-ARTI management.

## Conclusion

An acute home-based intervention embedded in a collaborative care network for patients with CA-ARTI requiring oxygen therapy is feasible and potentially cost-saving.

## Supplementary material

10.1136/bmjopen-2025-113288 online supplemental file 1

## Data Availability

Data are available upon reasonable request.
