# Supplementary material for: Is home-based management feasible for COVID-19 patients requiring oxygen therapy? A participatory action study and cost analysis
Source: BMJ Open. 2026 Sep 3;16(9):e113288. doi: 10.1136/bmjopen-2025-113288 (PMC13548275; doi:10.1136/bmjopen-2025-113288)
Supplement: Supplementary file 1 [file bmjopen-2025-113288.reviewer_comments.pdf]

PEER REVIEW HISTORY

BMJ Open publishes all reviews undertaken for accepted manuscripts. Reviewers are asked to complete a checklist review form and are provided with free text boxes to elaborate on their assessment. These free text comments are reproduced below.

ARTICLE DETAILS

Title (Provisional)

Is home-based management feasible for COVID-19 patients requiring oxygen therapy? A participatory action study and cost analysis

Authors

Boeijen, Josi A; van de Pol, Alma C; Smit, Karin; Van Uum, Rick T; Ahmad, Abeer; van der Meulen, Miriam P; van den Broek, Robert; Bijsterbosch, Wilma G; Venekamp , Roderick; Rutten, Frans H; Zwart, D.L; the Dutch General Practitioners Research Consortium, On behalf of

VERSION 1 - REVIEW

**Reviewer**	**1**
**Name**	**Rose, Suzanne J.**
**Affiliation**	**Stamford Hospital, Department of Research and Discovery**
**Date**	**23-Dec-2025**
**COI**	**None**

Thank you for the opportunity to review this manuscript.

In general, the article is in close alignment with the previously published article in this journal describing the design. The study itself did fall short by quite a bit of the planned enrollment. A key strength is the use of a participatory action research approach with a multidisciplinary expert panel, which allowed for the iterative evaluation and adjustment of the intervention. In addition, the design does make this approach potentially applicable to other settings and diagnoses.

Of note, only 13 patients were included, and only 10 fully received the intervention. This significantly limits the generalizability of the safety and clinical course data. The study also reported significant challenges in interprofessional communication and logistical bottlenecks, such as nursing shortages and delays in delivering oxygen concentrators. There is limited evidence for the type II patients, as one expired early on in the intervention, which created an even smaller data pool for that specific subgroup. As a pilot study, there is no direct comparison group, making the "cost-saving" estimates purely hypothetical and based

on historical/reference costs. It is somewhat concerning and should be addressed that some cost analysis was based on 'expert opinion'.

While the pilot study addresses a critical gap in acute home-based care, the current manuscript has limitations that must be addressed before publication:

1. Title: Please consider updating the title - it is somewhat confusing to read and understand. Perhaps the word 'of' between management and COVID-19 would be appropriate.

2. Introduction: The introduction is extremely short and could benefit from further details. Please provide appropriate reference(s) after the opening sentence.

This sentence is lacking references - "During the COVID-19 pandemic,

most initiatives focused on early discharge of admitted patients."

Articles that have addressed this and could be incorporated are:

https://pmc.ncbi.nlm.nih.gov/articles/PMC7384553/

https://pubmed.ncbi.nlm.nih.gov/33720552/

https://pmc.ncbi.nlm.nih.gov/articles/PMC10593172/

3. Safety Interpretation: The authors must more explicitly acknowledge that "feasibility" in a group of 10-13 patients does not equate to "safety" in a clinical sense. The safety data should be presented with a stronger caveat

4. Logistical Bottlenecks: The authors should provide more detail on how the "brief (<24h) hospital admission" to fix logistics impacts the definition of "home-based management" and whether this undermines the core goal of avoiding hospital admission.

5. Cost Analysis Clarity: The cost-saving estimates (€1087 to €2850) are a wide range. A more robust discussion on the "return on investment" and the patient volume required to offset the high set-up costs (€34,500 - €72,000) is necessary

6. Language and Proofreading: The manuscript contains several typos (e.g., "pademic," "hymodynamic," "licencing") that need correction

The manuscript provides a valuable blueprint for collaborative care, but the small sample size and significant logistical failures during the pilot mean it serves more as a "proof of concept" than a definitive study on home-based oxygen therapy.

**Reviewer**	**2**
**Name**	**Simonetti, Jacopo**
**Affiliation**	**Università Cattolica del Sacro Cuore**
**Date**	**28-Dec-2025**

**COI**	**I received fees from Chiesi Farmaceutici and Boehringer Ingelheim in the last 3 years.**

This manuscript by Boeijen J et al. synthesizes the results regarding safety, feasibility, and costs of a home-based intervention for COVID-19 patients requiring oxygen therapy. The scientific tone is appropriate, as well as the clarity of methods and limitations appear appropriately acknowledged. It will be of interest to both clinicians and researchers in respiratory medicine and appears suitable for publication on BMJ Open.

While the language is generally excellent; some minor corrections of typographical and stylistic errors should be needed. Below my observations:

- Page 4, line 3: check the typo in “pandemic”

- Page 6, line 25: check the typo in ““hemodynamic instability”

- Page 9, line 10: check the typo in “the remaining”

The implications for practice, in addition, are mainly discussed at a system and policy level. The authors may consider adding a brief, clinically oriented message highlighting what this study suggests for frontline clinicians when considering home-based management for hypoxemic patients.

**Reviewer**	**3**
**Name**	**Kelson, Zoe**
**Affiliation**	**University of Exeter, Mathematics**
**Date**	**14-Feb-2026**
**COI**	**None**

This pilot study aimed to explore safety, assess feasibility, and estimate cost of a home-based intervention for COVID-19 patients requiring oxygen therapy.

Reviewer comments:

"evaluated in a multidisciplinary expert panel using a participatory action research approach. Safety was evaluated by scrutinizing clinical course data and monitoring adverse events."

Can the authors please specify statistical methods in the Abstract and Methods?

"embedded in a regional collaborative healthcare network" [Abstract]

and

"Participants were included from November 2022 until December 2023 in Utrecht, the Netherlands."

Can the authors please comment on whether they deem the included sample of patients to be representative?

"Data on safety, feasibility and cost were collected during a 30-day follow-up period."

and

"Patient characteristics, symptomatology at inclusion and healthcare use data were extracted from the patients' GP electronic medical records (EMR). Clinical course data (vital parameters and symptom scores; Supplementary Table 2) were recorded by the monitoring team during remote monitoring contacts. Occurrence of serious adverse events(10), such as hospital admission or death, were reported directly to the research team by the involved healthcare professionals. The patients' GP EMR was reviewed for missed (serious) adverse events. WHODAS 2.0 (World Health Organization Disability Assessment Score 2.0)(11) data were collected by phone or visit on day 0 and 30. Patients scored their 'feeling of safety' on day 14 on a visual analogue scale (0-100; 0 = extremely unsafe, 100 = fully safe)."

and

"We collected communication data through structured evaluation forms for patient/informal caregiver and all involved healthcare professionals. Logistics data were collected through evaluation forms for the care coordination center, nursing care organization, oxygen supplier, and complemented with observations of the coordinating researcher. "

Can the authors please confirm if there was any missing data?

"Thirteen patients were included (77% female; mean age 68 years, standard deviation [SD] 18.2)."

Can the authors please provide a discussion on sample size?

"the intervention was piloted and iteratively evaluated in a multidisciplinary expert panel that assessed 12 pre-defined key elements related to safety and feasibility. "

Can the authors please clarify how many experts were involved, and how were they identified and invited?

Can the authors please assess if the expert panel can be considered to be representative of the wider population of experts?

"During short cycle evaluations the panel evaluated whether safety issues had occurred and if so, whether these were attributable to intervention elements. Subsequently, the panel established whether it was safe to proceed, or if any intervention adjustments were required. During large cycle evaluations, data from all included patients were re-evaluated"

and

"Feasibility was evaluated by scoring the pre-defined 'key elements'...

Scoring options were green (‘yes/sufficiently covered’); yellow (‘requires attention and/or minor adjustments’); red (‘failure of protocol and/or major/critical adjustment required); or grey (‘not applicable’). In the short cycle evaluations the research team completed a preliminary scoring based on the available data at that time. During the large evaluations the panel adjusted scores if needed."

Can the authors please describe how the expert panel reached agreement?

Did the authors consider following a Delphi process for consensus?

Can the authors please also clarify how the 12 elements were initially decided upon?

"We performed an explorative cost analysis evaluating the set-up costs and per patient intervention costs. We compared these costs to reference costs of a hospital admission using a mixed-method approach. The estimation of the set-up costs were based on expert opinion (Supplementary Table 3)."

Whilst sources and assumptions are tabulated, can the authors please also quantify and communicate uncertainty in cost estimates?

Furthermore, can the authors please include uncertainty in the analysis, and generate confidence intervals for each study outcome?

"The duration of admission was estimated in two scenarios, one assuming duration of admission to be equal to the length of days on oxygen and one assuming duration of admission to be equal to duration of the telemonitoring. "

Two valuable scenarios have been explored by the authors.

Can the authors please perform sensitivity analyses to explore the robustness of the study findings to alternative assumptions and estimations?

"Enrolled patients had a mean age of 68 years (SD 18) and 77% were female. Two patients lived in a care facility (15%) and the remaning in a private residence (Table 1). 23% of patients had no comorbidities; 38% had > 2 comorbidities. Nine patients were hypoxemic at inclusion (69%), and four had an increased respiratory rate (31%). "

Can the authors please consider undertaking subgroup exploratory analyses?

"Table 1: Participant characteristics (n = 13)."

Can all cohort demographics please be included here, such as sex, age, race or ethnicity, and BMI (as a continuous variable)?

"Table 2: Estimated costs per cost-component of the intervention on the first day and all subsequent days, compared to two scenarios of hospital admission costs "

Can the formulation of these costs please be detailed (e.g. providing equations in the supplementary material)?

"Thirteen detailed case studies yielded important information on feasibility. • Cost analysis was explorative due to retrospective data collection, but provides valuable insight into total potential cost-savings, and into elements that contribute to costs. • Since only ten patients fully received the intervention, safety data should be interpreted cautiously." [Strengths and Limitations]

and

"Some limitations deserve further attention. Firstly, since only 10 patients fully received the intervention, the clinical course data should be cautiously interpreted. Nonetheless, we believe the 13 detailed case studies yielded important information on safety and feasibility. Secondly, our pre-defined key elements may not be fully transferable to other contexts as the design of our collaborative network intervention remains at least partly context-related. Thirdly, the cost analysis was explorative due to retrospective data collection. Still, the results provide insight into total potential cost-savings, and into elements that contribute to costs. Moreover, the explorative cost calculation can guide design of a detailed prospective cost analysis of similar interventions."

The authors provide a discussion on the study limitations.

Thanks for providing a copy of the STROBE checklist.

VERSION 1 - AUTHOR RESPONSE

Reviewer 1

In general, the article is in close alignment with the previously published article in this journal describing the design. The study itself did fall short by quite a bit of the planned enrollment. A key strength is the use of a participatory action research approach with a multidisciplinary expert panel, which allowed for the iterative evaluation and adjustment of the intervention. In addition, the design does make this approach potentially applicable to other settings and diagnoses. Of note, only 13 patients were included, and only 10 fully received the intervention. This significantly limits the generalizability of the safety and clinical course data. The study also reported significant challenges in interprofessional communication and logistical bottlenecks, such as nursing shortages and delays in delivering oxygen concentrators. There is limited evidence for the type II patients, as one expired early on in the intervention, which created an even smaller data pool for that specific subgroup. As a pilot study, there is no direct comparison group, making the "cost-saving" estimates purely hypothetical and based on historical/reference costs. It is somewhat concerning and should be addressed that some cost analysis was based on 'expert opinion'. While the pilot study addresses a critical gap in acute home-based care, the current manuscript has limitations that must be addressed before publication:

Point 1: Title: Please consider updating the title - it is somewhat confusing to read and understand. Perhaps the word 'of' between management and COVID-19 would be appropriate.

Response 1: Thank you. We have revised the title to reflect the research question, design and setting more clearly.

"Is home-based management feasible for COVID-19 patients requiring oxygen therapy? An exploratory pilot study"

Point 2: Introduction: The introduction is extremely short and could benefit from further details. Please provide appropriate reference(s) after the opening sentence. This sentence is lacking references - "During the COVID-19 pandemic, most initiatives focused on early discharge of admitted patients." Articles that have addressed this and could be incorporated are:

https://pmc.ncbi.nlm.nih.gov/articles/PMC7384553/

https://pubmed.ncbi.nlm.nih.gov/33720552/

https://pmc.ncbi.nlm.nih.gov/articles/PMC10593172/

Response 2: Thank you. We have expanded the introduction and added the following references:

van Uum RT, Breteler M, Hardeman H, van de Pol AC, Smit K, Boeijen JA, Ahmad A, Rigter F, Venekamp RP, Rutten FH, Zwart DL. How Dutch initiatives to early discharge COVID-19 patients were organised during the pandemic: a scoping review. BMJ Open. 2025 Aug 26;15(8):e097839.

Point 3: Safety Interpretation: The authors must more explicitly acknowledge that "feasibility" in a group of 10-13 patients does not equate to "safety" in a clinical sense. The safety data should be presented with a stronger caveat.

Response 3: We agree with the reviewer that safety in a clinical sense cannot be concluded from 10-13 patients, and we decided to remove safety from the revised manuscript.

Point 4: Logistical Bottlenecks: The authors should provide more detail on how the "brief (<24h) hospital admission" to fix logistics impacts the definition of "home-based management" and whether this undermines the core goal of avoiding hospital admission.

Response 4: In our opinion, such a short stay in the hospital does not harm our 'home-based management' definition. This, because it was mostly for patients who were seen at the ED at the end of the day. This timing hampered logistics of arranging oxygen

concentrators at home, and nursing care at home. The advantage of allowing such a short stay is that some facilities and personnel did not need to be in operation 24/7 and that home-based management was blended to already existing hospital care and remote monitoring structures. With a very brief hospital stay, the chance of hospital-related complication or injury is much smaller than for a stay of at least three days (which is common for CA-ARTI).

Point 5: Cost Analysis Clarity: The cost-saving estimates (€1087 to €2850) are a wide range. A more robust discussion on the "return on investment" and the patient volume required to offset the high set-up costs (€34,500 - €72,000) is necessary.

Response 5: We agree that the uncertainty on the cost-saving estimates are large. As this paper is an exploratory analysis, we chose to also correctly display related uncertainty of cost-savings. The intervention itself was a natural experiment, thus without upfront data collection and a control group. Therefore, we do not know the length of stay if patients would have been admitted in the hospital instead. We chose to still illustrate potential cost-savings, while being transparent on the related uncertainty. Therefore, we presented scenarios with different length of stay in the hospital resulting in the range of cost-savings.

We agree that it would be useful to add the number of patients required to offset set-up costs and added the following paragraph in the results:

"With a cost-saving estimate of €1087 to €2850, and high set up costs of €34,500 - €72,000, 13 to 67 patients should undergo acute home management for CA-ARTI before the break-even point is reached."

Point 6: Language and Proofreading: The manuscript contains several typos (e.g., "pademic," "hymodynamic," "licencing") that need correction.

Response 6: Thank you, we corrected these in the revised manuscript.

The manuscript provides a valuable blueprint for collaborative care, but the small sample size and significant logistical failures during the pilot mean it serves more as a "proof of concept" than a definitive study on home-based oxygen therapy.

Reviewer 2

This manuscript by Boeijen J et al. synthesizes the results regarding safety, feasibility, and costs of a home-based intervention for COVID-19 patients requiring oxygen therapy. The scientific tone is appropriate, as well as the clarity of methods and limitations appear appropriately acknowledged. It will

be of interest to both clinicians and researchers in respiratory medicine and appears suitable for publication on BMJ Open.

Point 1: While the language is generally excellent; some minor corrections of typographical and stylistic errors should be needed. Below my observations:
Page 4, line 3: check the typo in “pandemic”
Page 6, line 25: check the typo in “hemodynamic instability”
Page 9, line 10: check the typo in “the remaining”

Response 1: We welcome the corrections and have adjusted the manuscript accordingly.

Point 2: The implications for practice, in addition, are mainly discussed at a system and policy level. The authors may consider adding a brief, clinically oriented message highlighting what this study suggests for frontline clinicians when considering home-based management for hypoxemic patients.

Response 2: We thank the reviewer for the constructive suggestion. We have added such a brief clinical message to the implications for practice section in the revised manuscript as follows:

“Further qualitative research is needed to identify the barriers and facilitators that influence healthcare professionals' willingness to participate in home-based management programmes. Concerns about increased workload are frequently cited, yet our experience suggests that, with well-organised logistics and central monitoring of oxygen saturation and clinical symptoms, the additional burden on both hospital physicians and GPs may be kept to a minimum.”

Reviewer 3

This pilot study aimed to explore safety, assess feasibility, and estimate cost of a home-based intervention for COVID-19 patients requiring oxygen therapy.

Point 1: *"evaluated in a multidisciplinary expert panel using a participatory action research approach. Safety was evaluated by scrutinizing clinical course data and monitoring adverse events."*
Can the authors please specify statistical methods in the Abstract and Methods?

Response 1: The aims of the study were to assess feasibility and estimate cost of home-based management, which are descriptive, exploratory goals. In other words, the study aimed

to test whether the intervention *can* work in practice, not to prove that it *does* work in a statistical sense. Moreover, we had no comparator group.

Feasibility and safety elements were evaluated by a multidisciplinary expert panel using a structured scoring system of predefined key points. This is in accordance with participatory action research methodology which does not rely in statistical inference. Participatory action research methodology prioritizes learning and iteration over statistical proof.

Point 2: *"embedded in a regional collaborative healthcare network" [Abstract] and "Participants were included from November 2022 until December 2023 in Utrecht, the Netherlands."*

Can the authors please comment on whether they deem the included sample of patients to be representative?

Response 2: Patients were representative for the Dutch population and included participants from urban and rural areas. We added the following sentence in the revised methods section:

"Participants were representative for the Dutch population and included participants from urban and rural areas."

Point 3: *"Data on safety, feasibility and cost were collected during a 30-day follow-up period."*

"Patient characteristics, symptomatology at inclusion and healthcare use data were extracted from the patients' GP electronic medical records (EMR). Clinical course data (vital parameters and symptom scores; Supplementary Table 2) were recorded by the monitoring team during remote monitoring contacts. Occurrence of serious adverse events (10), such as hospital admission or death, were reported directly to the research team by the involved healthcare professionals. The patients' GP EMR was reviewed for missed (serious) adverse events. WHODAS 2.0 (World Health Organization Disability Assessment Score 2.0)(11) data were collected by phone or visit on day 0 and 30. Patients scored their 'feeling of safety' on day 14 on a visual analogue scale (0-100; 0 = extremely unsafe, 100 = fully safe)."

"We collected communication data through structured evaluation forms for patient/informal caregiver and all involved healthcare professionals. Logistics data were collected through evaluation forms for the care coordination center, nursing care organization, oxygen supplier, and complemented with observations of the coordinating researcher."

Can the authors please confirm if there was any missing data?

Response 3: If applicable, missing data were reported alongside the reported results on specific outcomes in de Supplementary Tables 5 and 7. There were some missing data in the

WHODAS questionnaire, reported in Supplementary Table 5, and one case has missing data on GP consultation workload, reported in Supplementary Table 7.

Point 4: *"Thirteen patients were included (77% female; mean age 68 years, standard deviation [SD] 18.2)."*
Can the authors please provide a discussion on sample size?

Response 4: As described in the methods, this was a pilot study following a participatory action research approach focused on feasibility and costs with as purpose to test the practicability of an intervention, identify logistical and organizational barriers, and generate the evidence base needed to design a subsequent, adequately powered trial. Such studies are therefore not designed around a formal sample size calculation.
We acknowledge that 13 patients — and 10 patients who received the full intervention — is a small number, and we explicitly state that safety data are only exploratory given this limited sample. However, the detailed case-level data from these 13 patients were nonetheless sufficient to identify meaningful patterns in feasibility, pinpoint bottlenecks in communication and logistics, and generate plausible cost estimates. The iterative evaluation rounds conducted by the multidisciplinary expert panel allowed systematic learning from each case, which would not have been feasible — or necessarily improved — with a larger, less intensively monitored cohort.

We would also note that sample sizes in this range have been reported in participatory action research pilots before (1, 2).

(1) Pekmezaris R, Williams MS, et al. Adapting a home telemonitoring intervention for underserved Hispanic/Latino patients with type 2 diabetes: an acceptability and feasibility study. BMC Med Inform Decis Mak. 2020 Dec 7;20(1):324. doi: 10.1186/s12911-020-01346-0.

(2) Haesebaert J, Samson I, et al. How to engage patients in research and quality improvement in community-based primary care settings: protocol for a participatory action research pilot study. Res Involv Engagem. 2018 Oct 1;4:30. doi: 10.1186/s40900-018-0113-3.

Point 5: *"the intervention was piloted and iteratively evaluated in a multidisciplinary expert panel that assessed 12 pre-defined key elements related to safety and feasibility."*
Can the authors please clarify how many experts were involved, and how were they identified and invited?
Can the authors please assess if the expert panel can be considered to be representative of the wider population of experts?

Response 5: The process of recruitment of experts was previously described in the protocol paper.
Boeijen JA, van de Pol AC, van Uum RT, et al. Home-based management of hypoxaemic COVID-19 patients: design of the Therapy@Home pilot study. BMJ Open 2024;14:e079778. doi:10.1136/bmjopen-2023-079778.

We realize that a short recap is needed in current paper, and we added the following paragraph to the revised manuscript:

"The multidisciplinary expert panel was involved in the design, development, and evaluation of the intervention throughout. The panel was recruited by the research team from relevant stakeholders in the region(11). The panel consisted of GPs from four regional primary care groups; pulmonologists and acute care internists from four hospitals in the region; home care nursing organisations and an office for nursing care mediation; a monitoring centre; regional care coordination centre; and a patient representative from the Utrecht Elderly Care Network."

Point 6: *"During short cycle evaluations the panel evaluated whether safety issues had occurred and if so, whether these were attributable to intervention elements. Subsequently, the panel established whether it was safe to proceed, or if any intervention adjustments were required. During large cycle evaluations, data from all included patients were re-evaluated"*

"Feasibility was evaluated by scoring the pre-defined 'key elements'" [...] "Scoring options were green ('yes/sufficiently covered'); yellow ('requires attention and/or minor adjustments'); red ('failure of protocol and/or major/critical adjustment required); or grey ('not applicable'). In the short cycle evaluations the research team completed a preliminary scoring based on the available data at that time. During the large evaluations the panel adjusted scores if needed."

Can the authors please describe how the expert panel reached agreement?

Did the authors consider following a Delphi process for consensus?

Response 6: In the short-cycle biweekly evaluations, the research team first compiled and presented the available safety and feasibility data for the patients included since the previous evaluation. This included clinical course data, adverse event reports, and preliminary scoring of the relevant key elements by the research team based on direct feedback. Panel consensus agreement was reached through a structured, facilitated discussion in both the short (biweekly) and large (every 6–8 weeks) cycle discussions. We did not need formal voting.

We did not follow a formal Delphi process for consensus because it uses iterative rounds of written and (anonymous) feedback, minimizing dominant voice effects, achieving consensus over time. This is well-suited to situations where expert consensus needs to be established on certain recommendations, but not for scoring pre-defined 'key elements' per participant in real-time. The evaluation questions related to the 'key elements' were grounded in live clinical and organizational data, not on

expert opinion alone. The meetings were online or face-to-face and enabled the panel to respond rapidly to feasibility challenges, and to adjust the intervention accordingly.

Point 7: Can the authors please also clarify how the 12 elements were initially decided upon?

Response 7: The key elements were drafted by the researchers, guided by the Medical Research Council (MRC) framework for developing and evaluating complex interventions (*), as previously described in the protocol paper. The key elements were then evaluated and refined by the expert panel prior to the initiation of the pilot study.

(*) Skivington K, Matthews L, et al. A new framework for developing and evaluating complex interventions: update of Medical Research Council guidance. BMJ 2021;374:n2061. doi: 10.1136/bmj.n2061

Point 8: *"We performed an explorative cost analysis evaluating the set-up costs and per patient intervention costs. We compared these costs to reference costs of a hospital admission using a mixed-method approach. The estimation of the set-up costs were based on expert opinion (Supplementary Table 3)."*
Whilst sources and assumptions are tabulated, can the authors please also quantify and communicate uncertainty in cost estimates?

Response 8: In this type of bottom-up calculation, uncertainty itself would be based on assumptions as well. We are not conducting a cost analysis over a certain time period with individual patient data. Rather, we are performing a bottom-up calculation of intervention itself, by costing all elements of the structured care pathway designed to be the intervention. The number of healthcare activities (such as home care and GP visits) is not measured but based on the planned care pathway as well. All healthcare activities and personnel hours are multiplied with fixed unit costs, coming from either the guideline or salary scales. The majority of cost components does therefore not vary per patient. In practice or a different setting, costs can differ if the set-up of the intervention differ. However, this is due to adjusting the intervention instead of a representation of true uncertainty. We did present uncertainty around the estimates for implementation, as the hours spend were largely based on expert opinion.

Point 9: Furthermore, can the authors please include uncertainty in the analysis, and generate confidence intervals for each study outcome?

Response 9: Confidence intervals van only be generated using per patient estimates, while we based our cost estimates in the structured care pathways the intervention is based upon.

Point 10: *"The duration of admission was estimated in two scenarios, one assuming duration of admission to be equal to the length of days on oxygen and one assuming duration of admission to be equal to duration of the telemonitoring."*
Two valuable scenarios have been explored by the authors. Can the authors please perform sensitivity analyses to explore the robustness of the study findings to alternative assumptions and estimations?

Response 10: Indeed, we did some scenario analysis. A full health economic evaluation warrants also a deterministic and probabilistic sensitivity analysis. However, we did not perform a full health economic evaluation, but rather a bottom-up calculation of the costs of the intervention itself. Therefore, these standard health economic analysis techniques are not applicable for our calculation. We added text to the following sentences to further clarify the nature of the analysis:

"We performed an explorative cost analysis evaluating the set-up costs and per patient intervention costs using a mixed-method approach. As data collection was retrospective, we did not perform a full health economic evaluation, but rather a cost calculation of the intervention itself. To illustrate potential cost-savings, we did a comparison to reference costs of a hospital admission."

Point 11: *"Enrolled patients had a mean age of 68 years (SD 18) and 77% were female. Two patients lived in a care facility (15%) and the remaining in a private residence (Table 1). 23% of patients had no comorbidities; 38% had > 2 comorbidities. Nine patients were hypoxemic at inclusion (69%), and four had an increased respiratory rate (31%)."*
Can the authors please consider undertaking subgroup exploratory analyses?

Response 11: With 13 participants it is impossible to undertake subgroup analysis because the immense risk of chance findings. Moreover, this study was not designed to test hypotheses or detect differences between groups. It had descriptive and exploratory aims. In this context, subgroup analyses would be methodologically inappropriate.

Point 12: *"Table 1: Participant characteristics (n = 13)."*
Can all cohort demographics please be included here, such as sex, age, race or ethnicity, and BMI (as a continuous variable)?

Response 12: The demographics on sex and age are reported in the text. We have now added these demographics in Table 1 of the revised manuscript as requested.

We did not accrue data on race and ethnicity because it is in the Netherlands not allowed to use such data for research purposes.

We reported whether participants had obesity (BMI >30 kg/m2) because it is a recognized risk factor for severe COVID-19 and for complications during respiratory illness, and this threshold is routinely used in clinical risk stratification. We therefore chose to report BMI >30 kg/m² only, rather than reporting BMI as a continuous variable. Moreover, with a sample of 13 participants a mean BMI (SD) would not provide relevant additional information.

Point 13: "Table 2: Estimated costs per cost-component of the intervention on the first day and all subsequent days, compared to two scenarios of hospital admission costs."
Can the formulation of these costs please be detailed (e.g. providing equations in the supplementary material)?

Response 13: Unfortunately, before revising the legend of tabel 2, I would first need further clarification on what the reviewer would like to see.

"Thirteen detailed case studies yielded important information on feasibility. • Cost analysis was explorative due to retrospective data collection, but provides valuable insight into total potential cost-savings, and into elements that contribute to costs. • Since only ten patients fully received the intervention, safety data should be interpreted cautiously." [Strengths and Limitations]

"Some limitations deserve further attention. Firstly, since only 10 patients fully received the intervention, the clinical course data should be cautiously interpreted. Nonetheless, we believe the 13 detailed case studies yielded important information on safety and feasibility. Secondly, our pre-defined key elements may not be fully transferable to other contexts as the design of our collaborative network intervention remains at least partly context-related. Thirdly, the cost analysis was explorative due to retrospective data collection. Still, the results provide insight into total potential cost-savings, and into elements that contribute to costs. Moreover, the explorative cost calculation can guide design of a detailed prospective cost analysis of similar interventions."

The authors provide a discussion on the study limitations.

VERSION 2 - REVIEW

**Reviewer**	**1**
**Name**	**Rose, Suzanne J.**
**Affiliation**	**Stamford Hospital, Department of Research and Discovery**

Date 05-May-2026

COI

I feel the authors have adequately addressed the comments and concerns of all reviewers.
