# Supplementary material for: Is home-based management feasible for COVID-19 patients requiring oxygen therapy? A participatory action study and cost analysis
Source: BMJ Open. 2026 Sep 3;16(9):e113288. doi: 10.1136/bmjopen-2025-113288 (PMC13548275; doi:10.1136/bmjopen-2025-113288)
Supplement: Supplementary file 2 [file bmjopen-16-9-s001.docx]

**Supplementary Figure 1:** Eligible participants categorized into type I and type II patients.

**Caption**

Risk factors for a complicated disease trajectory: chronic obstructive pulmonary disease GOLD class III or IV, chronic lung condition managed by a pulmonologist, insulin-dependent/poorly controlled diabetes, immunocompromised condition, history of deep vein thrombosis or pulmonary embolism, severe heart failure (NYHA III/IV), renal insufficiency (eGFR <30ml/min/1.73 m^2^), liver failure (Child-Pugh B or C), severe obesity (BMI>35 kg/m^2^).

Abbreviations: ED = emergency department; GP = general practitioner.

**Supplementary Table 1**: in- and exclusion criteria for type I and type II patients

| **Adults with COVID-19 or CA-ARTI** | |
| --- | --- |
| **Inclusion criteria** | **Exclusion criteria** |
| *►*Symptoms are severe:   - Hypoxemia (SpO_2_ <94%), and/or - Respiratory distress (respiratory rate >24/min) | *►*Dementia or severe psychiatric illness which renders the patient unable to follow study instructions |
|  | *►*Known illness that prevents reliable pulse oximetry, e.g.:   - Severe anemia - Raynaud’s disease |
| *►*≥18 years of age |  |
| *►*Hemodynamically stable | *►*Clinical condition requires more elaborate care than can be organized at home^‡^ |
| *►*Support at home:   - Capable family member, or - Informal caregiver at home |  |
|  | *►*Severe comorbidities or risk factors^‡^   - COPD GOLD class III or IV - Chronic lung condition managed by a pulmonologist - Insulin-dependent/poorly controlled diabetes - Immunocompromised condition - History of deep vein thrombosis or PE - Severe heart failure (NYHA III/IV) - Renal insufficiency (eGFR <30ml/min/1.73 m^2^) - Liver failure (Child-Pugh B or C) - Severe obesity (BMI>35 kg/m^2^) |
| *►*Able to operate a pulse-oximeter |  |
| *►*Proficient in Dutch (or translation through informal caregiver) |  |
|  |  |
|  | *►*Patient and physician decide -based on shared decision- to withhold specific treatment altogether in end-of-life stage* |

^‡^ Exclusion criteria only applicable for type I patients

* Exclusion criteria only applicable for type II patients

*Abbreviations:*

*BMI = Body Mass Index; CA-ARTI = Community Acquired Respiratory Tract Infection; COPD = Chronic Obstructive Pulmonary Disease; eGFR = Estimated glomerular filtration rate; NYHA = New York Heart Association; PE = Pulmonary Embolism; SpO_2_ = peripheral oxygen saturation.*

**Supplementary Table 2**: Vital signs and measurements included in the remote monitoring routine

| **Measured three times daily** | |
| --- | --- |
| Oxygen saturation | Peripheral oxygen saturation [SpO_2_] (%) (after 5 minutes of rest) |
| Oxygen use | Liters per min |
| Heart rate | Beats per min |
| Temperature | Degrees of Celsius (ear thermometer) |
| Shortness of breath symptoms | Has shortness of breath increased since the last measurement? Yes/No |
| Shortness of breath score in rest | Scale from 0 (none) to 10 (extremely severe) |
| **Measured once daily** | |
| Coughing symptoms | Is there in increase in coughing symptoms since the last measurement? Yes/no |
| Coughing score | Scale from 0 (none) to 10 (extremely severe) |
| Physical condition | Are you feeling worse than yesterday? Yes/No |

**Supplementary Table 3**: Source and assumptions of calculation of costs of each component

|  | | **Source of resource use** | **Source of unit costs** | **Assumptions and remarks** |
| --- | --- | --- | --- | --- |
| **Set-up cost** | | | | |
|  | App development  App tailoring | Expert opinion | Expert opinion | Assumed 39 days of work, costing between €14.000 - €22.000. |
|  | App license | Expert opinion | Expert opinion | Costs depend on (already existing) licensing for other apps, estimated at €8000 - €30.000. |
|  | Infrastructure | Expert opinion | Expert opinion | Assumed 245 hours were spent. Total: €12.500 - €20.000 at assumed salary of €50 - €80 per hour. |
| **Per patient costs: first day^1^** | | | | |
|  | Ambulance ride | Observed in the study | Dutch costing guideline | An (planned) ambulance ride home.  [€273] |
|  | Care Coordination Centre | Expert opinion, based on internal reports | Expert opinion | Assumed 1.25 hour spent at 25 euros per hour.  [1.25 x €25 + 20% = €38] |
|  | Intake and instruction at ED | Expert opinion (1 person) on time spent | Expert opinion | Assumed 1.5 hour spent on intake and instruction at salary of 50 euros per hour.  [1.5 x €50 + 20% = €90] |
|  | Oxygen equipment delivery | Fixed costs | Oxygen supplier | Top-down fixed costs, including delivery costs, installation costs, rent, overhead.  [€250] |
|  | Nursing care intake | Fixed costs | Home care organization | Fixed costs of an intake (1.5 hour) and costs for personal hygienic materials added (€30).  [€186] |
|  | Monitoring center intake | Expert opinion (retrospective questionnaire) | Salary scale university medical centers; assumed % telephone contact | Assumed 20 minutes time spent per patient. Salary €15 per hour. Assumed 60% of time is spend for telephone calls.  [0.33* €15 / 0.6 + 20% = €12] |
|  | *Overhead* |  |  | *20% overhead was added to each component we calculated from personnel time.* |
| **Per patient costs: subsequent days^1^** | | | | |
|  | Nursing care | Expert opinion, assumed two or three visits | Fixed costs from home care organization | [ 2.5 visits x €41.80 = €105] |
|  | General practitioner visits | See Supplemental Table 7 | Dutch costing guideline | [(29 telephone, 6 physical and 1 email consultation * €30.87 + 5 home visits * €43.31) / 9 patients with information = €148] |
|  | Monitoring center | Expert opinion (retrospective questionnaire) on time spent per patient | Salary scale university medical centers. | Assumed length of each telephone contact minus intake length; Salary €15 per hour. Assumed 60% of time is spend for telephone calls.  [0.77 * €15 / 0.6 + 20% = €23] |
| **Comparison** | | | | |
|  | Hospital admission of 4.1 days | Based on study data of monitored patients (no controls available) | Dutch costing guideline^2^ | Assumed on length of oxygen therapy.  [4.1 days * €537 = €2202] |
|  | Hospital admission of 7.4 days | Based on study data of monitored patients (no controls available) | Dutch costing guideline^2^ | Assumed on length of remote monitoring.  [7.4 days * €537 = €3974] |

^1^ If applicable, personnel time was multiplied with a factor spent on patient contact (e.g. for telemonitoring) and multiplied with the salary scale estimated from expert opinion.

^2^ National Health Care Institute**.** Dutch Costing Guideline. <https://www.zorginstituutnederland.nl/over-ons/publicaties/publicatie/2016/02/29/richtlijn-voor-het-uitvoeren-van-economische-evaluaties-in-de-gezondheidszorg>; 2016.

**Supplementary Table 4:** Clinical course data of patients in home-based management (n = 10)

| **Duration of treatment** | | |
| --- | --- | --- |
|  | Number of days monitored mean (SD), [range] | 7.4 (3.4), [1-14] |
|  | Number of days with oxygen therapy, mean (SD), [range] | 4.1 (2.6), [0-7] |
|  | Number of days alive and out of hospital (30 days follow-up), mean (SD) | 25 (11.7) |
| **Clinical deterioration** | | |
|  | Clinical deterioration | 2* |
| **Medical treatment details** | | |
|  | Maximum oxygen supplementation (L/min) | 5 |
|  | Temperature >38 °C during home management (yes), n (%) | 1 (10%) |
|  | Antibiotics (yes), n (%) | 4 (40%) |
|  | Anticoagulants as thrombosis prophylaxis (yes)*, n (%) | 3 (30%) |

Abbreviations: SD = standard deviation.

*One patient was admitted to the hospital and subsequently died.

**Vitamin K antagonists or direct oral anticoagulants were chronic medication for 2/10 additional patients.

**Supplementary Table 5:** change in WHODAS 2.0^a^ disability score

|  | **Total** |
| --- | --- |
| Total WHODAS 2.0 score at baseline, mean (SD) | 27 (4.6) |
| Total WHODAS 2.0 score after 30 days, mean (SD) | 12 (5.0) |
| Percentage decrease after 30 days, mean (SD) | 54 (14.8) |

^a^The WHODAS 2.0 questionnaire includes 12 items rated on a five-point Likert scale (1-5); the total score is computed by summarising scores. Five represents no difficulties, 60 represents most severe difficulties.

Missings: 5 at day 0 and 6 at day 30.

Abbreviations: SD = standard deviation; WHODAS 2.0 = World Health Organization Disability Assessment Score 2.0.

**Supplementary Table 6**: detailed description of key elements that were scored red (‘failure of protocol and/or major/critical revision in protocol required’)

| a | The monitoring center could not reach the patient via phone. Informal caregiver refused contact after day 0. |
| --- | --- |
| b | The care coordination center forgot had forgotten to inform the monitoring team of inclusion of a new patient. The first contact moment with patient was missed. |
| c | The oxygen concentrator supplier was unsure of timely (<4 hours) delivery due to timing (Friday 5.30PM). |
| d | Nursing care on day 0, 1 and 2 was not possible due to personnel shortage (sick leave). Research physicians substituted nursing care. |
| e | Nurses were refused entry into the home by informal caregiver. |
| f | Nursing costs were not covered by insurance, because the patient resided in a care facility. |
| g | Measurements were not done or done only once (instead of 3 times) daily. More frequent measurements were not achievable for patient and informal caregiver. |
| h | Combination of a and g. |

**Supplementary Table 7**: Number of GP consultations in the 30-days after index contact for patients who received home-based management

|  | | **All patients***  n (%) | **Type I**  n (%) | **Type II**  n (%) |
| --- | --- | --- | --- | --- |
| **Total** | | 41 (100) | 17 (100) | 24 (100) |
| **Outside office hours** | | 2 (5) | 0 (0) | 2 (8) |
| **Type of contact** | | | | |
|  | Telephone | 29 (71) | 11 (65) | 18 (75) |
|  | Consultation at GP clinic | 6 (15) | 5 (29) | 1 (4) |
|  | Home visit | 5 (12) | 0 (0) | 5 (21) |
|  | E-mail | 1 (2) | 1 (6) | 0 (0) |
| **Initiator of contact** | | | | |
|  | GP | 22 (54) | 10 (59) | 12 (50) |
|  | Patient | 7 (17) | 6 (35) | 1 (4) |
|  | Informal caregiver | 8 (20) | 1 (56) | 7 (29) |
|  | Monitoring center | 4 (20) | 0 (0) | 4 (17) |
| **Reason for contact** | | | | |
|  | Planned follow-up | 23 (56) | 7 (41) | 16 (67) |
|  | New or deteriorating COVID-19 related symptoms | 6 (15) | 0 (0) | 6 (25) |
|  | Glucose regulation | 4 (10) | 3 (18) | 1 (4) |
|  | Post-recovery care | 5 (12) | 5 (29) | 0 (0) |
|  | Other | 3 (7) | 2 (12) | 1 (4) |

*Missing data for 1/10 patients.

Abbreviations: GP = general practitioner
